# Assessment of the usage and effectiveness of intermittent preventive treatment and insecticide-treated nets on the indicators of malaria among pregnant women attending antenatal care in the Buea Health District, Cameroon

**DOI:** 10.1186/s12936-016-1228-3

**Published:** 2016-03-17

**Authors:** Eric Bertrand Fokam, Leonard Ngimuh, Judith K. Anchang-Kimbi, Samuel Wanji

**Affiliations:** Department of Zoology and Animal Physiology, University of Buea, 63, Buea, Cameroon; Department of Microbiology and Parasitology, University of Buea, 63, Buea, Cameroon; Research Foundation in Tropical Diseases and Environment, P.O. Box 474, Buea, Cameroon

**Keywords:** IPT-SP, ITNs, Malaria, Pregnancy

## Abstract

**Background:**

Malaria in pregnancy is an immense public health problem with at least 50 million pregnant women living in malaria endemic areas. To prevent malaria and its complications in pregnancy the World Health Organization recommends the use of intermittent preventive treatment sulfadoxine–pyrimethamine (IPTp-SP), the use of insecticide-treated nets (ITNs), and effective case management. In most malaria endemic countries in Africa, 40 % of pregnant women sleep under ITNs. In Cameroon, about 90 % of pregnant women receive the first dose of SP, while 64 % take the complete dose. Following the 2011 mass-campaign of free distribution of ITNs coupled with routine ANC distribution of ITN and adoption of IPTp in Cameroon, little has been done to assess the effectiveness of both interventions outside of Yaoundé, the capital city. This study sought to assess the usage and effectiveness of IPTp-SP and ITNs on malaria in pregnancy.

**Methods:**

The research was a cross-sectional hospital-based study that included 410 pregnant women attending antenatal clinics in the Buea Health District. Capillary blood samples were collected to check malaria parasite by microscopy and haemoglobin levels by microhaematocrit technique.

**Results:**

A prevalence of 13.4 and 41.7 % was detected for malaria and anaemia, respectively. The Overall coverage of ITN was 32.4 % while that of ITPp was 63.2 %. Malaria prevalence was least (7.2 %) amongst women using both IPTp-SP and ITN while those with no intervention had the highest malaria prevalence of 18.6 % (χ2 = 6.188; P = 0.103). Of the women with malaria, 12.73 % were using ITN and had taken at least one dose of SP, 38.18 % had taken at least one dose IPTp only, 10.91 % were using only ITN and 38.18 % were not using any preventive measure. There was a difference in anaemia status within the different intervention groups (χ2 = 8.673; P = 0.034). Pregnant women using both interventions were less associated to malaria (OR = 0.341, 95 % CI = 0.138–0.841) compared to those using only one control method.

**Conclusion:**

Repeated doses of SP in combination with ITN use are effective in reducing malaria parasitaemia and improving haemoglobin level of pregnant women.

## Background

Malaria is a mosquito-borne disease of humans and other animals caused by parasitic protozoans of the genus *Plasmodium*. It is transmitted through the bite of an infected female *Anopheles* mosquito. According to the World Health Organization (WHO), an estimated 214 million new cases of malaria occurred worldwide in 2015 and 438,000 malaria deaths with the African region accounting for most global cases of malaria (88 %) [1].

Malaria in pregnancy is an immense public health problem with at least 50 million pregnant women living in malaria endemic areas [2]. Pregnant women are more likely than non-pregnant women to become infected with *Plasmodium falciparum* and, once infected, there is a tendency towards increased severity of disease [3, 4] caused in part by the transient depression of cell-mediated immunity that occurs during pregnancy [5].

Under conditions of high transmission, there is increased *P. falciparum* parasite density in placental and peripheral blood, which is higher in first, compared to later, pregnancies [6]. This epidemiological pattern for *P. falciparum* has been recognized for several years, with higher density in early pregnancy, which increases if untreated, and then decreases as gestation progresses [7]. In stable high transmission areas, almost all primigravidae, if unprotected, are likely to be infected in early pregnancy and approximately half of these would remain so by the time of delivery if untreated. In multigravidae, especially in those with higher parities, parasite density is significantly reduced due to the acquisition of parity-specific immunity [8]. The effects of malaria on pregnant women differ with various factors, such as the woman’s level of immunity, her gravidity, the trimester of pregnancy, and the presence or absence of co–morbidity [6, 9].

Malaria in pregnancy can have serious consequences for the mother, fetus, and newborn child, yet the harmful effects are preventable with interventions that are inexpensive and cost-effective and have been available for over two decades [10–12]. The WHO in 2004 recommended a package of intermittent preventive treatment in pregnancy (IPTp) with sulfadoxine-pyrimethamine (SP) and use of insecticide-treated nets (ITNs), together with effective case management of clinical malaria and anaemia.

In Cameroon, this policy was adopted and implemented by the Ministry of Public Health in 2004 immediately after WHO recommendations and consisted of at least three free SP doses between the 16th and the 36th weeks of pregnancy alongside use of ITNs. These interventions are commonly delivered in antenatal clinics (ANC) through collaboration between malaria and reproductive health programmes [1]. According to the world malaria report in 2011, in most malaria endemic countries in Africa, 40 % of pregnant women sleep under ITNs. In Cameroon, about 90 % of pregnant women receive the first dose of SP, while about 64 % take the complete dose [13]. Following the 2011 mass-campaign of free distribution of ITNs coupled with routine ANC distribution of ITN and adoption of IPTp in Cameroon little has been done to assess the effectiveness of these interventions with potentials of being efficacious, easy to administer, safe and tolerable [4, 12, 14], has not been fully evaluated together, so the combined effects of IPTp and ITN is still lacking information. This study was, therefore, designed to assess the usage and effectiveness of ITN and IPTp on malaria and anaemia in pregnancy. It was hypothesized that women using both IPTp plus ITN in pregnancy will be better protected against malaria and anaemia than women using only one of the prevention option (IPTp only, ITN only) or not using any at all.

## Methods

### Ethical considerations

Ethical clearance was obtained from the Institutional Review Board of the Faculty of Health Sciences at the University of Buea and administrative authorization obtained from the Regional Delegation of Public Health, South West Region and from the District Medical Officer of Buea; permission was sought from the different health facilities. Informed consent was obtained from the women prior to their interview and sample collection.

### Study area

The study was carried out in the Buea Health District (BHD) that comprises of both rural and urban communities. It has seven health areas with a total of 21 recognized health facilities. Six of whom were randomly selected using ballot (Mount Mary Hospital, Buea Road HC, Regional Hospital Buea, Solidarity Health Foundation, Molyko HC, Mile 16 IHC) from four health areas (Molyko, Buea Road, Muea and Bokwango).

### Study design and study population

The study was cross-sectional hospital-based and included all pregnant women attending antenatal services in the BHD. HIV positive women on cotrimoxazole were excluded to avoid confounder on IPT. A total of 410 pregnant women participated in the study from April to July 2014.

### Data collection

A simple structured questionnaire was used to interview participants in English; however, for all illiterate women, questions were translated into “Pidgin” (Creole). The questionnaire sought to obtain demographic data and data on IPTp-SP and ITN use. Hospital records of participants were used to confirm IPTp use and the dosage. The Roll Back Malaria (RBM) partnership indicator for ITN use, which is based on the proportion of pregnant women who slept under an ITN the previous night [15]), was equally utilized to determine ITN usage. The women were then placed into different groups; IPTp-SP plus ITN use, IPTp only, ITN only and no intervention. The term ITN in this study was referred to nets that had been treated with insecticide and needed ongoing treatment or long-lasting insecticide nets which are the most frequently distributed types of net in Africa [15]. A sample size of 408 had been estimated by the Watson formula [16] to provide the desired outcome at 5 % precision, 95 % confidence level and an estimated usage rate for IPTp of 90 % [13] and ITN 61 % [17] giving an average response rate of 75.5 %, that was used to calculate the sample size.

### Sample collection and processing

Blood samples were collected by finger prick after cleaning the finger with alcohol. A drop of blood was placed on a grease-free microscope slide and a thick blood film was made and stained for 15 min using 10 % Giemsa. More blood was collected in heparinized micro haematocrit tubes for determination of haemoglobin levels using packed cell volume (PCV). The Giemsa-stained thick smear were examined microscopically under 100X objective (oil immersion) using an Olympus microscope. Slides were considered positive when asexual forms and/or gametocytes of any Plasmodium species were observed on the blood films. A slide was declared negative after examining at least 100 high power field without any Plasmodium species, as described in [18, 19]. The microhaematocrit tubes were then centrifuged in a microhaematocrit centrifuge for 5 min at 10,000 rpm and read using the Hensin microhaematocrit reader according to the manufacturer instruction. PCV levels less than 30 % (10 g/dl) were classified as anaemia [20] since HB is a third of PCV.

### Data analysis

A template of the questionnaire was prepared using EpiInfo 3.5.3 and the data entered, cleaned and exported to SPSS version 20 (IBM SPSS, Chicago, IL, USA). Data was summarized into means and standard deviations and percentages were used in the evaluation of the descriptive statistics. Differences in Proportions were compared using the χ2 test. Means of Haemoglobin levels and malaria parasitaemia were compared using the Kruskall-Wallis test. Logistic regression was used to compare malaria and anaemia status in the different intervention groups with no intervention group as control or reference group. The level of significance was set to p < 0.05.

## Results

### Characteristics of study participants

A total of 410 pregnant women were enrolled among which 28 (6.8 %) were from Muea, 45 (11.0 %) from Molyko, 265 (64.5 %) from Buea Road and 72 (17.6 %) from Bokwaongo. The participants were aged 14–48 with a mean age of 26.54 ± 4.87. The gestation ages ranged from 8 to 41 weeks with a mean of 27.48 ± 6.95. Primigravidae constituted 24 % and multigravidae made 33.2 % of the study participants. Pregnant women in their third trimester were more likely to use IPTp (53.7 %) than those in the second trimester while women in their first trimester were less likely to use ITNs than the others. Women >25 years were more likely to use both intervention. More information about the demographic characteristics is displayed in Table 1.

**Table 1:** Demographic characteristics of study population

Factors	N = 410 (%)	ITN usage (133) (%)	IPTp usage (259) (%)
Age			
≤20	38 (9.3)	6 (4.5)	19 (7.3)
21–25	142 (34.6)	30 (22.6)	92 (35.5)
>25	230 (56.1)	97 (72.9)	148 (57.2)
Educational level			
Primary level	91 (22.2)	36 (27.1)	56 (21.6)
Secondary/high school	182 (44.4)	65 (48.9)	108 (41.7)
Tertiary	137 (33.4)	32 (24.1)	95 (36.7)
Gravidity			
Primigravid	152 (37.1)	28 (21.1)	94 (36.3)
Secundigravid	122 (29.8)	45 (33.8)	76 (29.3)
Multiplegravid	136 (33.2)	60 (45.1)	89 (34.4)
Occupation			
Students/unemployed	135 (33)	21 (15.8)	88 (34)
Business	123 (30)	43 (32.3)	79 (30.5)
Civil servant	76 (18.5)	35 (26.3)	50 (19.3)
House wife/farmer	76 (18.5)	34 (25.6)	42 (16.2)
Number of ANC visits			
First visit	88 (21.5)	25 (18.8)	5 (1.93)
Second visits	131 (32)	39 (29.3)	81 (31.3)
Three or more visits	191 (46.6)	69 (51.9)	173 (66.8)
Trimester			
First	19 (4.6)	5 (3.8)	0 (0)
Second	238 (58)	73 (54.9)	120 (46.3)
Third	153 (37.3)	55 (41.4)	139 (53.7)

### Coverage of IPTp and ITN

The coverage of ITN was 32.4 % while the coverage of ITPp was 63.2 %, with pregnant women greater than 25 years of age more likely to use both interventions. Of the 55 pregnant women with parasitaemia, 7 (12.73 %) were using ITN and had taken at least one dose of SP, 21 (38.2 %) had taken at least one dose IPTp, 6 (10.9 %) were using only ITN and 21 (38.2 %) used none of the interventions (SP or ITN).

### Prevalence of malaria and anaemia

**Fig. 1:**
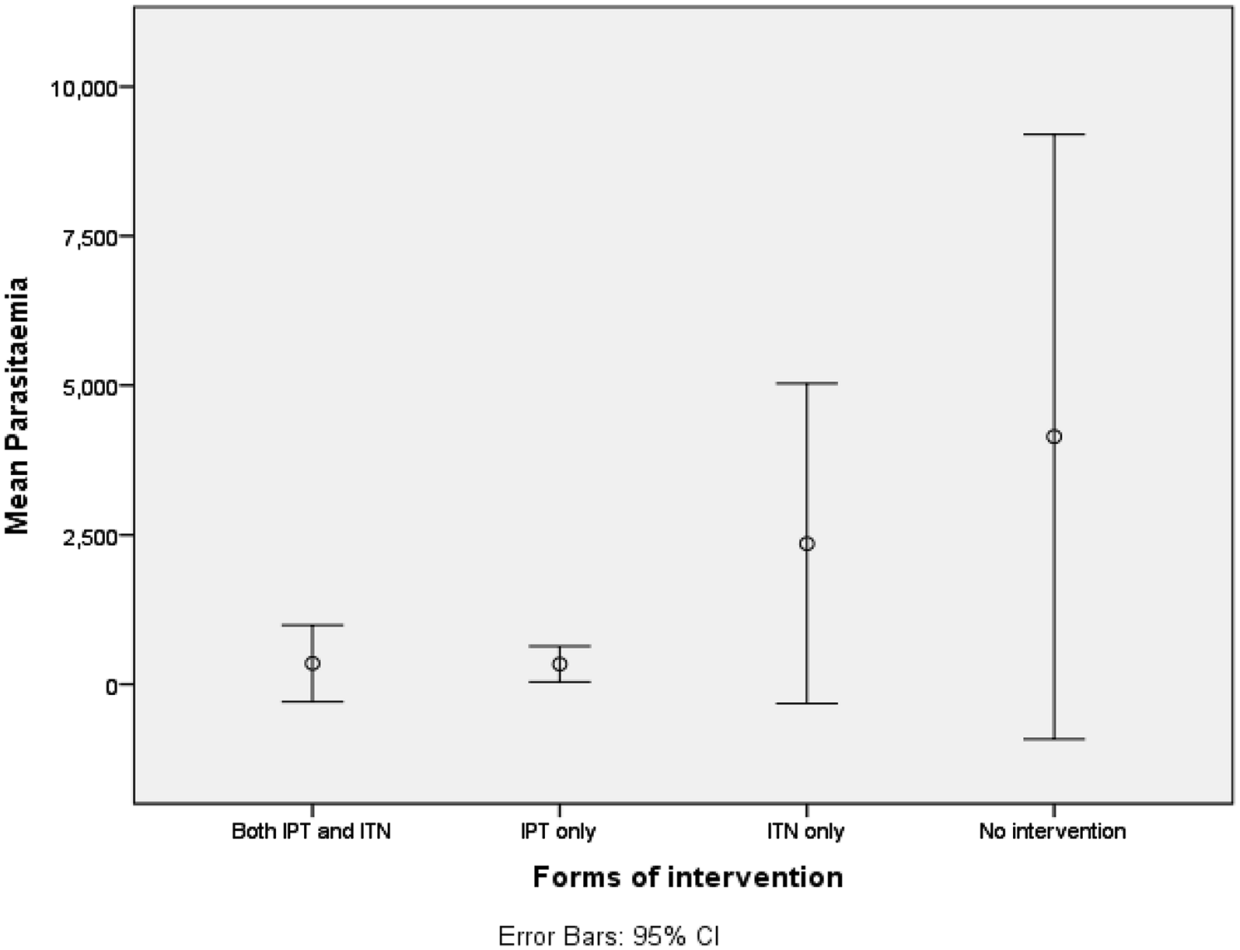
Influence of the different interventions on mean parasitaemia

**Fig. 2:**
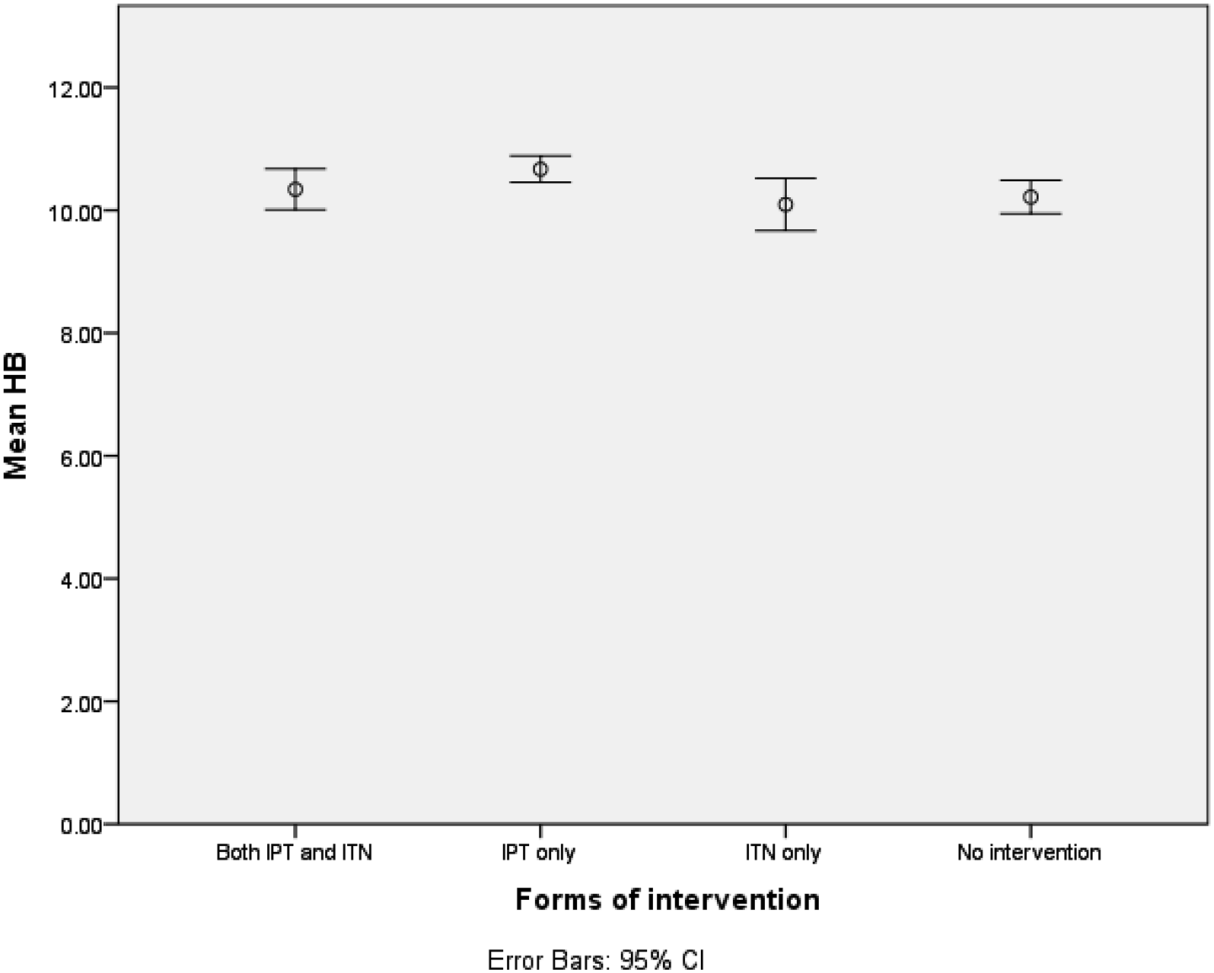
Effects of the different interventions on anaemia status

Out of the 410 women who participated in the study 55 (13.4 %) had malaria. Pregnant women attending ANC for the first time had a higher prevalence of malaria (18.2 %) though the difference was not statistically significant (χ2 = *2.335,* P = 0.311). Pregnant women using no malaria intervention were found to have the highest malaria prevalence (18.6 %) and mean parasite density (Fig. 1) compared to those using ITN only, IPTp only and both ITN and IPT (Table 2); the differences among them was not significant for both prevalence and mean parasitaemia, respectively (χ2 = *6.188, P* = 0.103 and χ2 = *6.973,* P = 0.073). The prevalence of anaemia was 41.7 %. The mean HB was 10.42 ± 1.48 and HB ranged from 6 to 15.70 g/dl. There was a significant difference in anaemia prevalence (χ2 = 8.673, P = 0.034) and mean haemoglobin levels (χ2 = 10.453, P = 0.015) in the different intervention (Fig. 2).

**Table 2:** Effects of the usage of the different interventions on malaria, parasitaemia and haemoglobin levels

Factor	Form of intervention				Total N = 410	Significance level
	IPT + ITN N = 97	IPT only N = 164	ITN only N = 36	None N = 113		
Malaria prevalence	7 (7.2 %)	21 (12.8 %)	6 (16.7 %)	21 (18.6 %)	55 (13.4 %)	χ2 = 6.188 P = 0.103
GMPD	123 ± 694	137 ± 653	915 ± 2549	461 ± 11,114	261 ± 7056	χ2 = 6.975 P = 0.073
Anaemia prevalence	46 (47.4 %)	54 (32.9 %)	17 (47.2 %)	54 (47.8 %)	171 (41.7 %)	χ2 = 8.673 P = 0.034
GMHB	10.34 ± 1.66	10.67 ± 1.40	10.10 ± 1.26	10.22 ± 1.47	10.42 ± 1.48	χ^2^ = *10.453* P = 0.015

GMPD is geometric mean of parasite density and GMHB is geometric mean of Haemoglobin levels

### Effects of the usage of IPT-SP and ITN on malaria prevalence and haemoglobin levels

Those without intervention (Table 2) had the highest malaria prevalence (18.6 %), highest GMPD (461 ± 11114) while those using only ITN had the least GMHB with a significant difference within the groups (χ^2^ = *10.453*, P = 0.015).

### Effects of IPT-SP and ITN on malaria prevalence

**Fig. 3:**
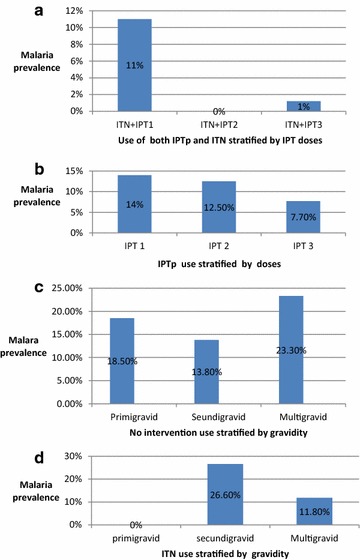
Effects of IPT-SP and ITN on malaria prevalence

Pregnant women with at least two doses of SP and using ITN are better protected than women with just one SP dose in addition to ITN use (Fig. 3a), likewise, women using only IPT-SP have least malaria prevalence (7.70 %) with three successive doses of SP.

### Effectiveness of IPT and ITN on malaria control

Using no intervention as control and comparing it with ITN, IPT and IPT + ITN (Table 2), those using IPT combined with ITN were less associated to malaria (OR = 0.341, CI = 0.138–0.841), followed by those using IPT only (OR = 0.643, CI = 0.333–1.244) while those using only ITN were more associated to malaria (OR = 0.876, CI = 0.323–2.373).

## Discussion

Malaria and anaemia in pregnancy are important public health problems in the Buea Health District (BHD). These problems contribute significantly to high maternal and child mortality. It is well known that pregnant women living in many parts of sub-Saharan Africa have an increased risk of malaria infection, but limited data have been reported on the impact of IPTp and ITN in combination for pregnant women in Cameroon. The goal of this study was to assess the usage the impact of IPTp-SP and ITN use on malaria in pregnant women in the BHD.

A prevalence of 13.4 and 41.7 % was recorded for malaria and anaemia respectively. The prevalence for malaria was lower than the 32.7 % reported in 2005 [21] in a similar area. This reduction as the years go by may be due to the systematic free distribution of bed nets and IPT-SP to pregnant women. The prevalence of anaemia was lower than that reported earlier in other areas of the country [13, 22]. This could be due the free distribution of iron, folic acid and other blood giving supplement to pregnant women attending ANC, coupled with the fact that the coverage of IPTp-SP is on the rise in the country where at least 90 % of pregnant women have been shown to receive the first dose [13], which can reduce malaria-related anaemia.

The coverage of ITN was 32.4 % which less than what was reported in Sudan and Tanzania [17, 23] with main reasons advanced by the women for not using nets being hot weather, absent of mosquito, in conveniences which is similar to some of the reasons seen in a meta analysis [12]. The coverage of ITPp was 63.2 % which is less than that reported in Sudan [17] with main reason being late ANC start.

In this study, malaria prevalence was higher in the IPTp-SP plus ITN group at first SP dose compared to those at second and third dose (Fig. 3a). This could be attributed to the effectiveness of SP drug to clear most parasitaemia in the pregnant women and the additional protection provided by bed nets, which has also been shown in the meso-endemic area of the Thai-Burmese border [24] and in the Gambia [25] to reduce maternal malaria and anaemia. The slightly higher prevalence of malaria in pregnant women who took three successive doses of SP in addition to ITN use could be as a result of possible development of resistance of the parasites to SP in some women and also may be due to the long interval between SP doses. The was a systematic reduction in prevalence of malaria with increasing doses of SP with those using only IPTp (Fig. 3b) which shows the effectiveness and impact of SP in malaria control. Multigravid women using only ITN had the highest malaria prevalence (Fig. 3c) than secundigravid and primigravid women, this is different from other studies which found primigravid women were more susceptible due to the production of antibodies in successive pregnancies that prevent parasite sequestration in the placenta [26].

Those not using any intervention and those using only ITN had the least geometric mean of HB (Table 2 and Fig. 2) with a significant different in GMHB (χ^2^ = *10.453,* P = 0.015) within the different interventions simply because most of those in this group were attending ANC late and for their first time and therefore had not received any of the supplements given to pregnant women to prevent anaemia.

This study also shows that pregnant women not using any intervention had the highest geometric mean of parasite density. This could be due to the lack of any barrier or prophylactic measure to prevent malaria, such as ITN and IPTp whose effectiveness and efficacy has been shown [27]. Those using both forms of interventions were less associated to malaria compared to the other intervention groups (OR = 0.341, 95 % CI = 0.138–0.841). This is probably because of the ability of SP to clear malaria parasite and the effectiveness of ITN to prevent malaria though SP might have cover up for inconsistent use of ITN. This could be confirmed from the fact that those using IPT only were less associated to malaria than those using ITN only (OR = 0.876, 95 % CI = 0.323–2.373).

## Conclusion

There was a decrease in peripheral parasitaemia with improved HB levels following increasing doses of SP/IPTp in addition to the use of ITN. The use of both interventions (IPT +ITN) reduces malaria morbidity compared to using a single intervention. Sensitization of pregnant women on the combined used of ITNs and IPT-SP during pregnancy would be pivotal to achieving significant results in the struggle to reduce malaria-related complication in pregnancy. This would be achieved by encouraging pregnant women to attend ANC early during pregnancy, in order to be drilled on the benefits of taking complete doses of SP. This study will help public health authorities and actors to undertake mass awareness campaigns to educate mothers on the importance of using both IPT and ITN during pregnancy.
